# Environmental Preconditioning Shapes the Expression and Post-Formulation Stability of Plant Growth-Promoting Traits in Native Actinobacteria

**DOI:** 10.3390/polym18172041

**Published:** 2026-08-22

**Authors:** María Elena Mancera-López, Josefina Barrera-Cortés

**Affiliations:** Zacatenco Unit, Biotechnology and Bioengineering Department, Center for Research and Advanced Studies of the National Polytechnic Institute, Mexico City 07360, Mexico; elenamlopez@hotmail.com

**Keywords:** alginate encapsulation, physiological plasticity, bioinoculants, UV resistance

## Abstract

The functional expression of plant growth-promoting (PGP) traits in soil actinobacteria is conditioned by abiotic factors, yet the combined effects of pH and temperature on their metabolic profiles and the stability of these profiles after encapsulated formulation and post-processing stress remain insufficiently characterized. This study aimed to evaluate the physiological plasticity of native actinobacteria and the expression of plant growth-promoting (PGP) traits under different pH and temperature conditions, as well as their stability after encapsulation, dehydration, and exposure to UV irradiation. Strains isolated from a semi-arid agricultural soil were analyzed to determine their ability to produce indole-3-acetic acid (IAA), siderophores, and phosphatases, as well as their ability to fix nitrogen, degrade cellulose, and tolerate salt stress. Temperature and pH significantly affected all evaluated PGP traits (*p* < 0.001), and their expression was not directly associated with biomass production. Two strains, S1 and S4, exhibited the highest overall PGP indices. Strain S1 maximized IAA and siderophore production under neutral conditions (pH 7.0, 30 °C), whereas strain S4 maintained more stable phosphatase activity across the tested pH and temperature ranges. Cell viability remained above 85% after encapsulation and dehydration. Dehydration enhanced IAA and siderophore production in strain S1, while strain S4 exhibited transient metabolic activation under UV irradiation in non-dehydrated capsules. The encapsulation matrix preserved cell viability more effectively than it preserved the complete PGP functional profile, indicating that viability alone is an insufficient criterion for evaluating the technological success of alginate-based bioinoculant formulations. These findings highlight the importance of integrating environmental preconditioning and functional stability assessments into the development of robust microbial bioinoculants adapted to agricultural systems subjected to fluctuating environmental conditions.

## 1. Introduction

The intensive use of inorganic fertilizers and pesticides since the 1960s has substantially increased agricultural productivity. However, their underutilization and excessive application have contributed to soil degradation, nutrient losses, groundwater contamination, and ecosystem deterioration worldwide [1,2,3]. In Mexico, these practices have also raised concerns regarding long-term food security and environmental contamination [4,5].

Plant growth-promoting microorganisms (PGP-Ms) have emerged as a sustainable alternative to reduce dependence on synthetic fertilizers and mitigate the environmental impacts associated with conventional agricultural practices [6]. Such microorganisms enhance plant development through both direct and indirect mechanisms. Direct mechanisms include improving nutrient acquisition, modulating phytohormone levels, and increasing tolerance to abiotic stresses, whereas indirect mechanisms involve suppressing phytopathogens through the production of antimicrobial compounds and competition for nutrients and ecological niches [6,7,8]. Among these microorganisms, actinobacteria are particularly attractive due to their extensive metabolic diversity, widespread distribution in agricultural soils, and remarkable capacity to synthesize bioactive compounds of agronomic interest, making them promising candidates for the development of multifunctional bio-inputs [9,10,11]. Despite growing interest in PGP-Ms, only a few commercial products based on actinobacteria are currently available, including Mycostop^®^ (*Streptomyces griseoviridis* K61, Espoo, Finland) and Actinovate^®^ (*Streptomyces lydicus* WYEC 108, Milwaukee, WI, USA), both of which rely partly on siderophore and hydrolytic enzyme production—PGP mechanisms directly evaluated in the present study [12].

Actinobacteria synthesize a wide range of metabolites associated with plant growth promotion, including indole-3-acetic acid (IAA), siderophores, phosphatases, and hydrolytic enzymes such as cellulases and chitinases. These compounds stimulate root development, increase the availability of nutrients such as phosphorus and iron, and contribute to the suppression of soilborne phytopathogens, thereby improving plant health and productivity [6,9,11]. The simultaneous expression of multiple PGP traits within a single strain may confer greater functional robustness under fluctuating environmental conditions, highlighting the importance of evaluating these traits in an integrated manner and under abiotic gradients representative of agricultural soils [10,13].

The production of microbial metabolites is strongly influenced by environmental factors such as soil pH and temperature [14]. Soil pH affects the solubility and bioavailability of essential nutrients, including phosphorus and iron, as well as the stability and activity of extracellular enzymes [15]. Temperature, in turn, modulates enzyme kinetics, membrane fluidity, and overall metabolic activity [16]. Several studies have demonstrated that changes in these parameters can significantly alter the production of PGP metabolites in actinobacteria, either positively or negatively, depending on the cultivation conditions [16,17]. Variations in metabolite production under contrasting environmental conditions can be interpreted as an expression of physiological plasticity, defined as the ability of an organism to modify its functional phenotype in response to environmental fluctuations without undergoing permanent genetic changes [18,19]. In PGP-Ms, this attribute may determine the consistency of beneficial functions under heterogeneous soil conditions and therefore represents a potentially useful criterion for strain selection.

Immobilization in calcium alginate matrices and controlled drying have been widely employed to improve the handling, storage, and application of microbial bioinoculants by providing mechanical protection, reducing water activity, and extending formulation shelf life [12,20,21]. In particular, calcium alginate gels prepared with a high mannuronic-to-guluronic acid ratio, as previously characterized by ^1^H NMR for the alginate source used in this study, form softer and more elastic “egg-box” junctions with Ca^2+^, a structural property associated with greater capsule flexibility and resistance to cracking during dehydration [12]. Nevertheless, preservation of cell viability does not necessarily guarantee maintenance of PGP functions, since these activities depend on the integrity of metabolic pathways that may be impaired during formulation and storage processes [12,20,22]. In addition, bioinoculants applied to seeds, plant surfaces, or exposed soils are frequently subjected to ultraviolet (UV) radiation, which can induce DNA damage, oxidative stress, and enzyme inactivation, thereby compromising not only cell survival but also metabolic functionality [23].

Although several studies have examined the effects of pH and temperature on plant growth-promoting traits in actinobacteria, comparatively few have evaluated their interactive effects using factorial experimental designs [14,17]. Likewise, although alginate encapsulation has been widely adopted for microbial formulation, its impact on maintaining metabolic functionality following drying and UV exposure has received considerably less attention than cell survival [12,20,21,22]. Furthermore, it remains unclear whether physiological plasticity expressed under environmental gradients translates into enhanced functional stability after formulation [19,24]. Determining whether this attribute can serve as an objective criterion for selecting robust strains represents an unresolved methodological issue that limits the rational development of bioinoculants for heterogeneous agricultural environments.

Therefore, this study aimed to determine the influence of pH and temperature on the physiological plasticity and expression of PGP traits in actinobacteria isolated from agricultural soils and to assess whether encapsulation in calcium alginate preserves metabolic functionality and cell viability following drying and ultraviolet irradiation. We hypothesized that pH and temperature differentially modulate the expression of PGP traits in native actinobacteria and that strains exhibiting greater physiological plasticity across environmental gradients retain higher levels of metabolic functionality after encapsulation, drying, and UV exposure. The results are expected to contribute to the rational design of robust, functionally stable, and effective microbial bio-inputs for agricultural systems exposed to environmental variability.

## 2. Materials and Methods

### 2.1. Isolation of Actinobacteria from Soil Samples

Actinobacterial strains were isolated from an agricultural soil collected in Nextlalpan, Edo. Mexico (19°41′32.4′′ N 99°04′39.4′′ W), of characteristics reported in Vera-García et al. (2023) [25]. Isolation was performed by serial dilution and spread plating. Briefly, 1 g of soil was suspended in 9 mL of sterile distilled water and serially diluted up to 10^−5^. Aliquots (100 µL) from the 10^−2^ to 10^−5^ dilutions were spread onto Czapek Dox^®^ agar (Condalab, Torrejón de Ardoz, Madrid, Spain) plates and incubated at 30 °C for 10 days. After incubation, six colonies displaying morphological characteristics commonly associated with actinobacteria were selected based on macroscopic features, including colony consistency, texture, shape, size, pigmentation of the aerial and substrate mycelia, diffusible pigment production, and a characteristic earthy odor [26,27]. Selected isolates were purified through successive subculturing on Czapek Dox^®^ agar (Condalab, Torrejón de Ardoz, Madrid, Spain) until morphologically uniform cultures were obtained. The six purified isolates were designated S1–S6 and used in all subsequent analyses. For short-term preservation, sporulated cultures were flooded with sterile distilled water and gently scraped to recover the spores [26]. The resulting suspension was filtered to remove mycelial fragments, transferred to sterile 1.5-mL microcentrifuge tubes, and stored at 4 °C until use.

### 2.2. Inoculum Preparation

Inocula were prepared from actinobacterial isolates grown on oatmeal agar (ISP3) [27]. Agar plugs (approximately 0.5 × 0.5 cm) containing actively growing mycelium were aseptically excised and used to inoculate 125-mL Erlenmeyer flasks containing 50 mL of ISP3 broth previously sterilized in an autoclave (Hirayama Hiclave HV-50, Amerex Instruments, Lafayette, CA, USA) at 121 °C for 15 min.

The cultures were incubated at 30 °C and 200 rpm in an orbital shaker (Thermo Forma 420, Forma Scientific Inc., Marietta, OH, USA) for approximately 72 h, until abundant growth was obtained. Culture purity was verified by subculturing aliquots onto Czapek Dox^®^ agar plates, followed by microscopic examination (40×) using an Olympus CH30RF100 microscope (Olympus, Tokyo, Japan).

All inocula used in subsequent experiments were prepared under identical cultivation conditions to minimize physiological variability among strains.

### 2.3. Experimental Design

The expression of plant growth-promoting (PGP) traits in actinomycetes depends on environmental conditions that regulate both primary metabolism and the biosynthesis of secondary metabolites [14]. Based on this premise, a full factorial experimental design (6 × 3 × 3) was implemented, including six actinomycete strains (S1–S6), three pH levels (5.5, 7.0, and 8.5), and three incubation temperatures (25, 30, and 37 °C) [14,17].

The experimental workflow consisted of two sequential stages: (i) physiological conditioning of inocula under defined pH–temperature combinations, and (ii) evaluation of morphological, physiological, and biochemical traits under standardized assay conditions. For each strain × pH × temperature combination, two independent biological replicates were prepared in separate experimental runs, with each independently prepared culture considered an experimental unit. Evaluated traits included indoleacetic acid (IAA) production, siderophore production, nitrogenase activity, phosphatase activity (acid and alkaline), chitinase activity, cellulolytic capacity, and salt tolerance. The assays for each trait were conducted independently using the corresponding culture medium and experimental conditions.

### 2.4. Morphological Characterization of the Strains

Morphological characterization was performed to identify phenotypic variability among isolates under different culture conditions.

#### 2.4.1. Macroscopic Characterization

Colony morphology was evaluated after 7 days of incubation on Czapek Dox^®^ agar at 30 °C. Diffusible pigment production, colony color, texture, shape, and the development of aerial and substrate mycelium were recorded by direct observation of cultures in solid and liquid phases.

#### 2.4.2. Micromorphological Characterization

Micromorphological traits were analyzed in ISP3 broth under controlled pH and temperature conditions (as defined in the experimental design). Cultures were incubated until stable growth was achieved.

Microscopic observations were performed at 40× magnification (Olympus CH30RF100, Tokyo, Japan), and the following parameters were evaluated: Pellet size and density, mycelial fragmentation, hyphal dispersion in liquid medium, and sporulation intensity. Traits were scored using a semi-quantitative ordinal scale (0–3) developed for this study, where increasing values indicated higher intensity or abundance.

Strain identity was additionally confirmed by 16S rRNA gene sequencing for strains S2–S6, which were identified as *Streptomyces* sp. (GenBank accession numbers PZ784602–PZ784606, respectively); identification of strain S1 is currently in progress. Colony morphology and molecular identification details for all strains are provided in Supplementary Table S1.

#### 2.4.3. Data Treatment

Morphological data were treated as ordinal variables and used for comparative phenotypic assessment among strains.

### 2.5. Qualitative Analysis of Plant Growth-Promoting Traits

Qualitative and semi-quantitative assays were performed to evaluate the functional potential of actinomycete strains under solid culture conditions. All assays were incubated at 30 °C using the pH conditions specific to each culture medium.

#### 2.5.1. Nitrogen Fixation Ability

Nitrogen fixation potential was assessed on nitrogen-free bromothymol blue (NFB) medium. Cultures were inoculated by stabbing (10 µL) and incubated for 14 days. Growth and colony expansion were used as indirect indicators of nitrogen fixation capacity and classified into three categories based on colony diameter [28]: low (0 < colonies ≤ 5 mm), medium (5 mm ≤ colonies ≤ 10 mm), and abundant (colonies ≥ 10 mm).

#### 2.5.2. Phosphate Solubilization

Phosphate solubilization was evaluated on Pikovskaya agar (PVK) [29]. Formation of clear halos around colonies was recorded after 7 days. The phosphate solubilization index (PSI) was calculated according to [30], an approach that continues to be applied in recent studies on phosphate-solubilizing bacteria [31].

(1) $$PSI=\frac{colony\;diameter\left(cm\right)+halo\;diameter\;(cm)}{colony\;diameter\;(cm)}$$

PSI ≤ 1.0, no activity; 1.5 ≤ PSI < 2.0, low activity; 2.0 ≤ PSI < 3.0, moderate activity; 3.0 ≤ PSI, high activity; PSI = 0, no microbial growth.

#### 2.5.3. Cellulolytic and Chitinolytic Activity

Cellulolytic activity was evaluated on carboxymethylcellulose (CMC) agar as described by Teather & Wood (1982) [32]. Chitinolytic activity was evaluated on a mineral medium supplemented with 1% (*w*/*v*) colloidal chitin as the sole carbon source [33]. After incubation, hydrolysis halos were visualized by flooding plates with Congo red (0.1%, *w*/*v*) for 15 min, followed by destaining with 1 M NaCl. The hydrolysis index (HI) was calculated as follows [34,35].

(2) $$HI=\frac{halo\;diameter\;(cm)}{colony\;diameter\;(cm)}$$

HI = 1, no activity; 1 < HI < 2, moderate enzymatic activity; HI ≥ 2, good enzymatic activity.

### 2.6. Quantitative Analysis of Secondary Metabolites and Enzymatic Activities

#### 2.6.1. Inoculum Preparation and Culture Conditions

Standardized inocula (1.8 × 10^8^ ± 2.5 × 10^7^ CFU mL^−1^) were prepared from ISP3 cultures conditioned under the experimental design. Cultures were grown in ISP3 medium (10 mL) adjusted to the corresponding pH and incubated at 25, 30, or 37 °C with agitation (200 rpm) for 96 h. The corresponding inoculum was prepared for each strain.

*Streptomyces lydicus* (CDBB1232), acquired from the National Collection of Microbial Strains and Cell Cultures at Cinvestav, was included as a reference strain.

#### 2.6.2. Indoleacetic Acid (IAA) Production

IAA production was quantified using the Salkowski method in L-tryptophan-supplemented medium. After incubation, cultures were centrifuged, and supernatants reacted with Salkowski reagent [36,37]. Absorbance was measured at 530 nm, and concentrations were determined using a calibration curve (0–50 µg mL^−1^).

#### 2.6.3. Siderophore Production

Siderophore production was quantified using the colorimetric iron perchlorate method [38], adapted for actinobacteria by Franco-Correa et al. (2010) [39]. Absorbance was measured at 480 nm, and concentrations were estimated using a desferrioxamine standard curve.

#### 2.6.4. Phosphatase Activity

Acid and alkaline phosphatase activities were determined using *p*-nitrophenyl phosphate (*p*-NPP) as substrate [39]. Reactions were incubated for 30 min at 30 °C and stopped with NaOH. Absorbance was measured at 405 nm, and activity was expressed as U mg^−1^ protein. One unit (U) of phosphatase activity was defined as the amount of enzyme that catalyzed the formation of 1 μmol of *p*-nitrophenol per minute under the assay conditions [40]. Total protein was quantified by the Bradford colorimetric method [41].

#### 2.6.5. Indirect Estimation of Nitrogen Fixation by Ammonium Production

Nitrogenase activity was estimated indirectly by ammonium production using the phenol–hypochlorite method [42]. Absorbance was measured at 640 nm, and nitrogenase activity was calculated from a standard curve prepared with ammonium chloride standard expressed as U mg^−1^ protein. One unit (U) was defined as the amount of enzyme that catalyzed the formation of 1 μmol NH_4_^+^ per minute under the assay conditions.

#### 2.6.6. Chitinase Activity

Chitinase activity was determined by using colloidal chitin as substrate and quantifying reducing sugars via the DNS method [43]. Absorbance was measured at 540 nm, and activity was expressed as U mL^−1^ based on N-acetylglucosamine calibration.

#### 2.6.7. Salt Tolerance

Salt tolerance was evaluated in ISP9 medium supplemented with NaCl (2.5, 5.0, and 7.5% *w*/*v*), following procedures commonly used to evaluate halotolerance in PGP actinobacteria [44]. Biomass was determined gravimetrically after drying at 60 °C. Final biomass was expressed as g L^−1^.

### 2.7. Selection Criteria: Plant Growth-Promoting Global Index (PGP-GI)

The selection of strains with the highest PGP potential was performed using a multi-criteria integration approach. Each strain was scored on a semi-quantitative scale (1–5; where 5 represents the highest performance) for the following traits: indoleacetic acid (IAA) production, siderophore production, acid phosphatase activity, alkaline phosphatase activity, nitrogenase activity, and salinity tolerance, considering all temperature and pH combinations evaluated in the experimental design.

Each trait was weighted according to its reported physiological relevance in plant–microbe interactions [6]. IAA and siderophore production were assigned the highest weight (20% each), whereas phosphatase and nitrogenase activities were assigned intermediate weights (18% each). Salinity tolerance was incorporated as a complementary stress-response trait.

The PGP-GI was calculated as:

(3) $$PGI-GI=\sum \left(w_{i}\times S_{i}\right)$$

where w_i_ represents the weight assigned to each trait, and S_i_ the normalized score.

The two strains with the highest PGP-GI values were selected for encapsulation and further formulation.

### 2.8. Encapsulation and Formulation of Selected Strains

Selected strains were propagated in ISP3 medium under the pH and temperature conditions identified as optimal for metabolite production. Immobilization was performed using calcium alginate via the extrusion method with calcium chloride as the crosslinking agent [12]. The mannuronic/guluronic acid composition of this alginate source was previously determined by ^1^H NMR for the same encapsulation system [12] and was therefore not redetermined in the present study, which focused instead on the strain-dependent functional stability of the encapsulated PGP traits.

Encapsulated formulations were subjected to controlled dehydration and subsequent ultraviolet (UV) exposure assays. Viability and metabolic activity were evaluated at each processing stage (encapsulation, dehydration, and UV exposure). Capsule diameter was measured using ImageJ software (v1.54g, NIH, Bethesda, MD, USA).

All experiments were performed in duplicate under sterile conditions.

#### 2.8.1. Encapsulation by Extrusion in Calcium Alginate Matrix

Strains were cultivated in 400 mL of ISP3 medium under optimal conditions (200 rpm, 96 h) starting from a standardized inoculum (1.8 × 10^8^ ± 2.5 × 10^7^ CFU mL^−1^). Biomass was concentrated by sedimentation and resuspended in 1.5% (*w*/*v*) sterile sodium alginate solution prepared in ISP3 medium at the corresponding pH.

The alginate-cell suspension was extruded dropwise using a peristaltic pump PCS PP 101-1 (Process Control Systems AG, Wetzikon, Switzerland) and sterile needles (31G × 8 mm and 20G × 9.5 mm) into 400 mL of sterile 0.2 M CaCl_2_ under gentle agitation (50 rpm, 1 h, 25 ± 2 °C).

Formed beads were washed, collected by filtration, and stored in sterile distilled water at 4 °C. Capsule diameter (2.5 ± 0.5 mm) was controlled by maintaining a constant extrusion distance. Yield was 100 ± 10 g of wet capsules per 400 mL suspension.

Viability was determined by CFU counting after release of cells using 1% sodium citrate. Metabolic activity was assessed by incubating capsules in saline solution (0.85%) followed by quantification of biomass, IAA, siderophores, and enzymatic activities as described in Section 2.6.

#### 2.8.2. Dehydration of Encapsulated Biomass

Wet capsules (33 ± 4.5 g) were dehydrated in sterile glass columns (0.65 L; 6 cm × 23 cm) with filtered airflow (4 L min^−1^) at 25 ± 2.5 °C. Air was sterilized using membrane filtration and porous stone diffusion.

The process was terminated when outlet relative humidity reached 20%. Capsule diameter decreased to approximately 1 mm. Samples were stabilized at 30 °C for 24 h before analysis [12].

Water loss was determined gravimetrically. Viability and metabolic activity were evaluated using 0.01 g of dry capsule samples following the methods described in Section 2.6.

#### 2.8.3. UV Photoprotection Assay (365 nm)

Samples were exposed to UV-A radiation (365 nm) generated by a UVGL-25 lamp (4 W, UVP, USA) positioned 7 cm above the samples. Based on the manufacturer’s nominal irradiance of 760 μW cm^−2^ at 7.6 cm, the estimated doses received after 3 and 6 h of exposure were approximately 9.7 and 19.3 J cm^−2^, respectively, conditions comparable to field UV-A exposure levels [23]. After exposure, 0.01 g of samples were collected for viability (CFU) and metabolic activity assays (IAA, siderophores, and enzyme activities). All experiments were performed in duplicate under sterile conditions.

### 2.9. Statistical Analysis

Statistical analyses were performed using IBM SPSS Statistics v18 (IBM Corp., Armonk, NY, USA). A three-way ANOVA was used to evaluate the effects of strain, temperature, and pH on biomass production, followed by Tukey’s HSD post hoc test (*p* < 0.05).

For continuous positive response variables (IAA, siderophores, phosphatases, nitrogenase activity, and phosphate solubilization index), generalized linear models (GLMs) with a Gamma distribution and log-link function were applied, including main effects and interaction terms (two- and three-way). Model fit was evaluated using deviance/df and Pearson χ^2^/df ratios. In cases of underdispersion (values < 1), the scale parameter was estimated using the deviance method to obtain robust standard errors [45,46]. Pairwise comparisons were performed using estimated marginal means with Bonferroni correction.

For ordinal or non-normally distributed variables (NFB growth, cellulolytic and chitinolytic activity, and salinity tolerance), the Kruskal–Wallis test was applied, followed by Mann–Whitney U tests with a Bonferroni-adjusted significance level (α = 0.005).

Statistical significance was defined as *p* < 0.05.

## 3. Results

### 3.1. Microbial Growth in Solid and Liquid Culture

Six strains exhibiting typical actinomycete morphological characteristics (Table 1) were isolated. Analysis of strains S1–S6 indicated that most preferred neutral pH (7.0) for growth, although marked differences in stress tolerance were observed. Strain S1 was the most sensitive, showing a 73.9% reduction in colony size under both acidic (pH 5.5) and alkaline (pH 8.5) conditions. In contrast, strains S2 and S3 were more robust, with S3 exhibiting a reduction of only 12.5% under alkaline conditions. Strains S4 and S6 maintained relatively stable growth across all pH conditions; however, S4 consistently formed small, slow-growing colonies (~1.0 cm), whereas S6 produced large, vigorous colonies (≥2.1 cm). Strain S5 exhibited an alkaliphilic tendency, deviating from the general trend. Acidic conditions strongly inhibited its growth (0.5 ± 0.2 cm), whereas alkaline conditions promoted growth, reaching a maximum colony size of 0.9 ± 0.3 cm at pH 8.5.

Distinct pH-dependent morphological and pigmentary changes were also observed. At pH 8.5, pigment production was induced in S1, and sporulation-associated alterations were observed in S3. Slightly acidic conditions (pH 5.8) induced central dark pigmentation in S2 and S6, whereas S4 produced pigment only under near-optimal conditions (pH 6.8), with pigment production being suppressed under stress conditions.

Semi-quantitative evaluation of morphological features of strains cultivated in ISP3 broth under controlled pH conditions at 30 °C is presented in Table 2. The parameters assessed included pellet size and density, mycelial fragmentation, and sporulation capacity. Pellet size and compaction varied among strains and pH conditions, as did the release of hyphae and spores into the culture medium. In general, pellets were more compact under acidic conditions (pH 5.5), whereas greater cell dispersion was observed at neutral and alkaline pH values. Strain S1 released spores at pH 7.0, while strains S3 and S5 exhibited hyphal release under neutral and alkaline conditions. Strain S6 showed the greatest morphological variability, forming small pellets accompanied by the simultaneous release of hyphae and spores at pH 5.5. These observations indicate that pH influences mycelial morphology and the stability of cell aggregates, thereby modulating the tendency of the strains to disperse in liquid culture. The release of hyphae and spores may represent an adaptive response associated with changes in pellet organization under different pH conditions.

### 3.2. Biomass Production

The ANOVA assessing the effects of temperature, pH, and strain on biomass production revealed significant effects of temperature and pH, as well as a significant temperature × pH interaction (F_temperature_ = 18.117, df = 2, *p* < 0.001; F_pH_ = 3.273, df = 2, *p* = 0.044; F_temperature × pH_ = 2.64, df = 4, *p* = 0.042). In contrast, the strain factor did not have a significant effect (Fstrain = 0.836, df = 6, *p* = 0.546). According to the data presented in Table 3, biomass production was highest in cultures incubated at 37 °C, with a mean value of 9.24 ± 1.06 g L^−1^. However, this yield was 8.7% lower than that of the control strain (*Streptomyces lydicus*). Regarding pH, the highest biomass production was observed under acidic conditions (pH 5.5), reaching 8.91 ± 0.86 g L^−1^. This value was also lower than the control’s, though by a smaller margin (3.7%).

### 3.3. Qualitative Assays of Plant Growth-Promoting Activity

#### 3.3.1. Growth of Strains in a Nitrogen-Free Medium

The Kruskal–Wallis test was applied to evaluate the effects of strain, pH, and temperature on microbial growth, treated as an ordinal variable. The analysis showed that growth in NFB medium was mainly influenced by strain identity (χ^2^ = 25.519, *p* < 0.001) and temperature (χ^2^ = 7.182, *p* = 0.028), whereas pH had no significant effect (χ^2^ = 1.693, *p* = 0.429) (Table 4). Overall, strains exhibited clear differences in growth capacity under identical conditions, with S4 showing the highest growth and S5 the lowest. Regarding temperature, higher growth was observed at 25–30 °C, while growth was significantly reduced at 37 °C. These results suggest that moderate temperatures favor the metabolic activity of the isolates, whereas elevated temperatures limit their development.

Multiple pairwise comparisons among strains and temperature levels were performed using the Mann–Whitney U test with Bonferroni correction (α = 0.05). Significant differences were observed between S4 and S1, S5, and S6 (*p* < 0.001), as well as between S5 and S2, S3, S4, and *Streptomyces lydicus* (*p* = 0.002). No significant differences were detected in the remaining comparisons.

#### 3.3.2. Phosphate Solubilization

Growth on Pikovskaya medium was influenced by the culture conditions used during inoculum propagation. Strain S5 failed to grow under any of the evaluated conditions. In contrast, strains S1, S3, and S6 exhibited reduced growth when inocula were produced at 37 °C compared with lower propagation temperatures, indicating sensitivity to elevated temperatures. Conversely, strains S4 and *S. lydicus* maintained growth across the evaluated conditions, showing no apparent inhibitory effects associated with changes in pH or temperature.

The phosphate solubilization index (PSI) for each evaluated strain is presented in Table 5. Overall, phosphate-solubilizing activity was primarily observed in strains S4 and *S. lydicus*, which exhibited low to moderate PSI values, with the highest performance recorded when inocula were propagated at 25 °C. Strains S1, S3, and S6 also displayed phosphate-solubilizing activity, although their PSI values were considerably lower and were restricted to specific propagation conditions.

The PSI data were analyzed using a generalized linear model (GLM) with a gamma distribution and a log-link function, considering only conditions in which microbial growth was observed (PSI > 0). The model yielded low values for both the dispersion parameter (deviance/df = 0.021) and the Pearson χ^2^ statistic (Pearson χ^2^ = 0.019), indicating an adequate fit to the experimental data. Analysis of model effects revealed significant influences of strain (Wald χ^2^ = 358.019, *p* < 0.001) and temperature (Wald χ^2^ = 39.069, *p* < 0.001) on PSI. However, these results should be interpreted with caution because of the potential for model overfitting.

#### 3.3.3. Cellulolytic Activity

The results of cellulolytic activity, expressed as the hydrolysis index on CMC-containing solid medium, are presented in Table 6. The Kruskal–Wallis test revealed a significant effect of temperature on cellulolytic activity (χ^2^ = 39.193, *p* < 0.001). Subsequent pairwise comparisons using the Mann–Whitney U test showed significant differences between 25 and 30 °C (*p* < 0.001) and between 25 and 37 °C (*p* < 0.001). Maximum cellulolytic activity was observed at 30 °C for most of the evaluated strains (S1, S2, S3, S4, and S6). In contrast, cellulolytic activity was markedly reduced at 25 °C and was virtually undetectable in all strains except S2. Increasing the temperature from 30 to 37 °C resulted in a decrease in cellulolytic activity in strain S2, whereas the remaining strains maintained activity levels comparable to those observed at 30 °C.

Overall, the results indicate that cellulolytic activity was strongly influenced by temperature, with the highest hydrolysis indices generally recorded at 30 °C. Differences among strains were also observed, particularly regarding their ability to maintain cellulolytic activity under suboptimal temperature conditions.

#### 3.3.4. Chitinolytic Activity

Microbial growth was observed on chitin-containing medium; however, no chitin degradation halos were detected. Therefore, the methodology used did not provide evidence of extracellular chitinolytic activity. Consequently, colony diameter on colloidal chitin agar was recorded as the only quantifiable parameter (Table 7). The Kruskal–Wallis test applied to microbial growth data, considering strain, temperature, and pH as factors, revealed significant effects of strain (χ^2^ = 19.405, *p* = 0.004) and temperature (χ^2^ = 11.360, *p* = 0.003). In general, growth of all strains was observed when cultures were prepared using inocula produced at 30 °C. In contrast, no growth was observed for strains S4, S5, and *S. lydicus* when inocula were produced at 25 °C, nor for strains S1, S5, and S6 when inocula were produced at 37 °C.

Based on colony diameter, microbial growth followed the order S2 > S3, S4 > S1, S6, *S. lydicus* > S5, with colony diameters ranging from 0.5 to 2.0 cm. Overall, the results indicate that the strains were able to establish growth on chitin-enriched medium; however, no detectable extracellular degradation of the polymer was observed under the conditions evaluated.

### 3.4. Plant Growth-Promoting Metabolites

#### 3.4.1. Indole-3-Acetic Acid (IAA)

**Figure 1:**
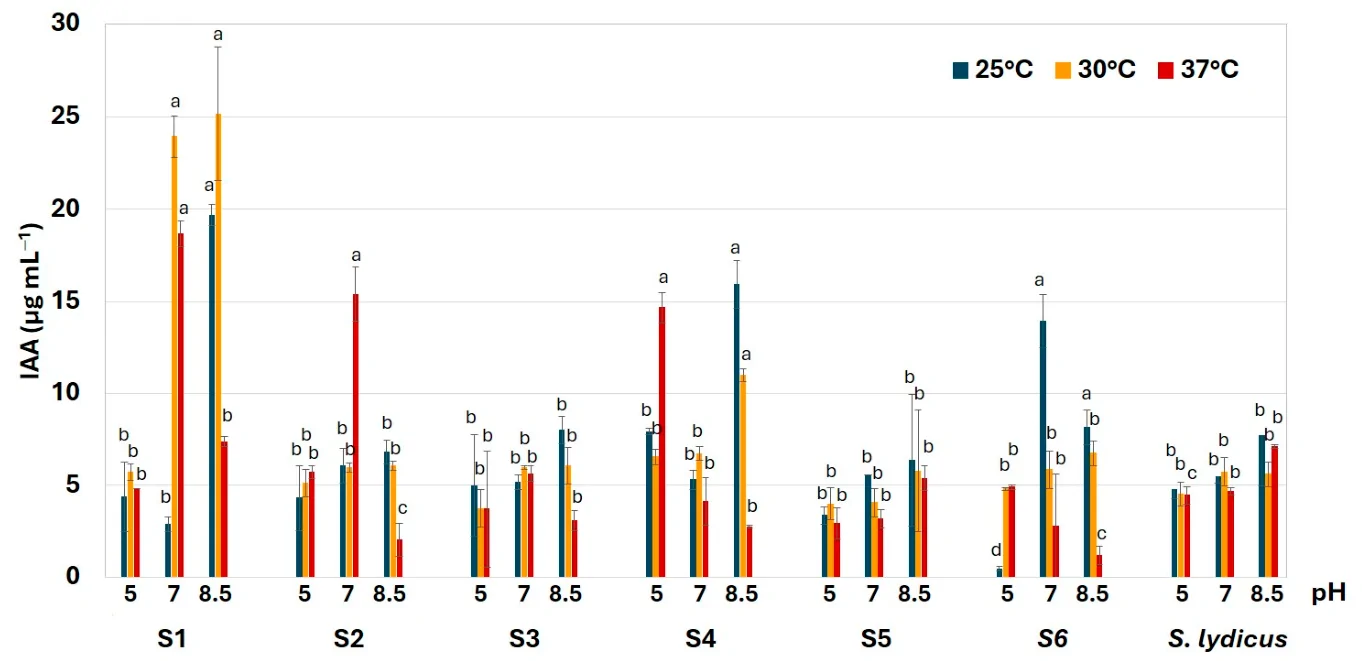
Effect of inoculum propagation conditions (pH and temperature) on indole-3-acetic acid (IAA) production by actinomycete strains. Bars labeled with different letters differ significantly (*p* < 0.05), as determined by pairwise comparisons of estimated marginal means using a generalized linear model with a Gamma distribution and log link; bars sharing at least one letter do not differ significantly.

A generalized linear model (GLM) with a Gamma distribution and log-link function revealed that indole-3-acetic acid (IAA) production was significantly affected by temperature (χ^2^ = 11.538, df = 2, *p* < 0.003), pH (χ^2^ = 19.654, df = 2, *p* < 0.001), and strain (χ^2^ = 47.652, df = 6, *p* < 0.001), as well as by the interaction among these factors (χ^2^ = 83.866, df = 24, *p* < 0.001). These results indicate that the response of IAA production to environmental conditions was strongly strain-dependent (Figure 1).

Compared with the control strain, S1 exhibited the highest IAA production, reaching levels 100–300% greater under neutral to alkaline conditions (pH 7.0–8.5) and temperatures of 30–37 °C. Strain S4 also showed high IAA production, followed by strains S2 and S6, although their responses were restricted to a narrower range of pH and temperature conditions.

#### 3.4.2. Siderophores

**Figure 2:**
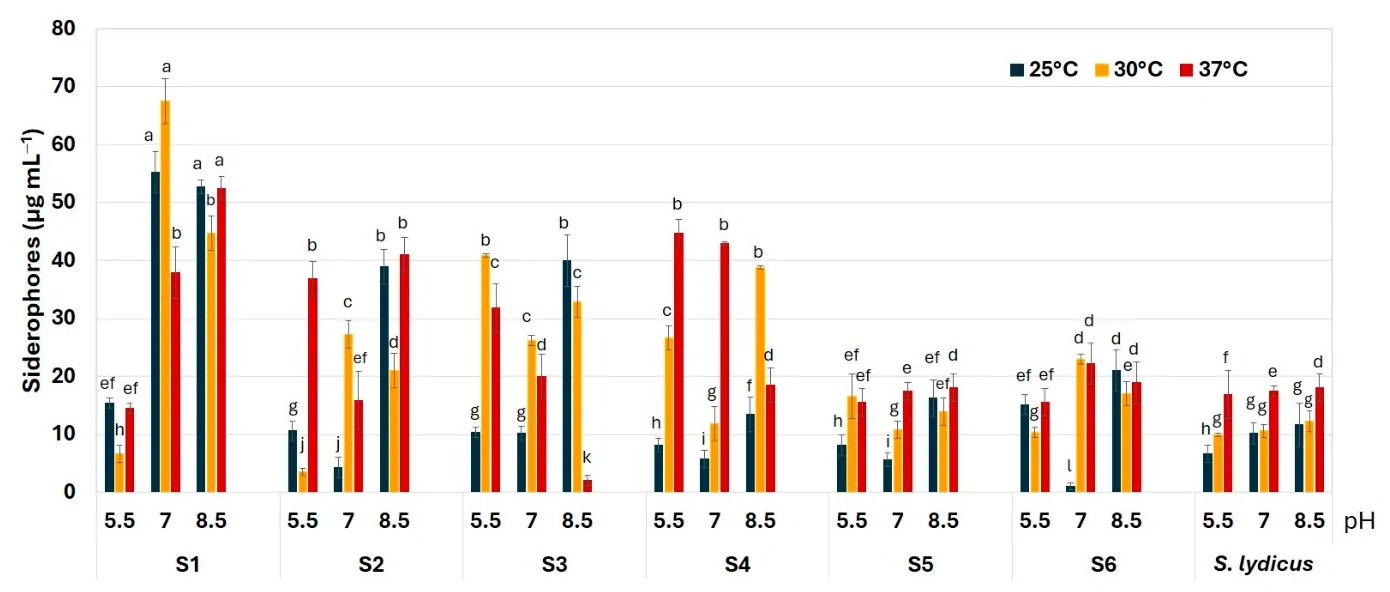
Siderophore production by strains S1–S6 under different temperature and pH conditions. Bars labeled with different letters differ significantly (*p* < 0.05), as determined by pairwise compar-isons of estimated marginal means using a generalized linear model with a Gamma distribution and log link; bars sharing at least one letter do not differ significantly.

Analysis of the main effects (temperature, pH, and strain) and their interactions using a generalized linear model (GLM) with a Gamma distribution and log-link function showed that temperature (χ^2^ = 437.141, df = 2, *p* < 0.001), pH (χ^2^ = 253.894, df = 2, *p* < 0.001), and strain (χ^2^ = 718.156, df = 6, *p* < 0.001) had significant effects on siderophore production (Figure 2). In addition, all two-way interactions, as well as the three-way interaction (temperature × pH × strain), were statistically significant (χ^2^ = 1258.631, df = 24, *p* < 0.001). These results indicate a strong multifactorial dependence of siderophore production, suggesting that optimization of the process requires the simultaneous consideration of culture conditions and strain-specific variability.

Overall, increased temperature and pH were associated with higher siderophore production. Among the evaluated strains, S1 exhibited the highest mean production (38.6 µg mL^−1^). Strains S2, S3, and S4 showed intermediate production levels (approximately 38–46% lower than S1), whereas S5 and S6 showed the lowest production (approximately 58–64% lower than S1).

The deviance/df and Pearson χ^2^/df ratios were considerably lower than 1 (0.032), suggesting potential underdispersion. This behavior may be associated with model complexity resulting from the inclusion of multiple interaction terms, which could indicate model overfitting. Notably, removal of the three-way interaction term improved model performance, increasing the deviance/df and Pearson χ^2^/df ratios from 0.032 to 0.237 and 0.185, respectively.

#### 3.4.3. Alkaline and Acid Phosphatase Activity

The factors temperature, pH, and strain, as well as their two-way interaction (temperature × pH), showed significant effects on alkaline and acid phosphatase activities, according to a generalized linear model (GLM) with a gamma distribution and log-link function (*p* < 0.05). Dispersion diagnostics indicated initial underdispersion, which was assessed using the deviance method (alkaline phosphatases: deviance/df = 0.331; Pearson χ^2^/df = 0.277; acid phosphatases: deviance/df = 0.226; Pearson χ^2^/df = 0.202).

Alkaline and acid phosphatase production increased significantly (Wald χ^2^ = 2286.417, *p* < 0.001; and Wald χ^2^ = 3123.890, *p* < 0.001, respectively) when the temperature was changed from 25 to 30 °C, reaching approximately 15-fold and 9-fold higher activity, respectively. A further increase to 37 °C resulted in inhibition of phosphatase production.

**Figure 3:**
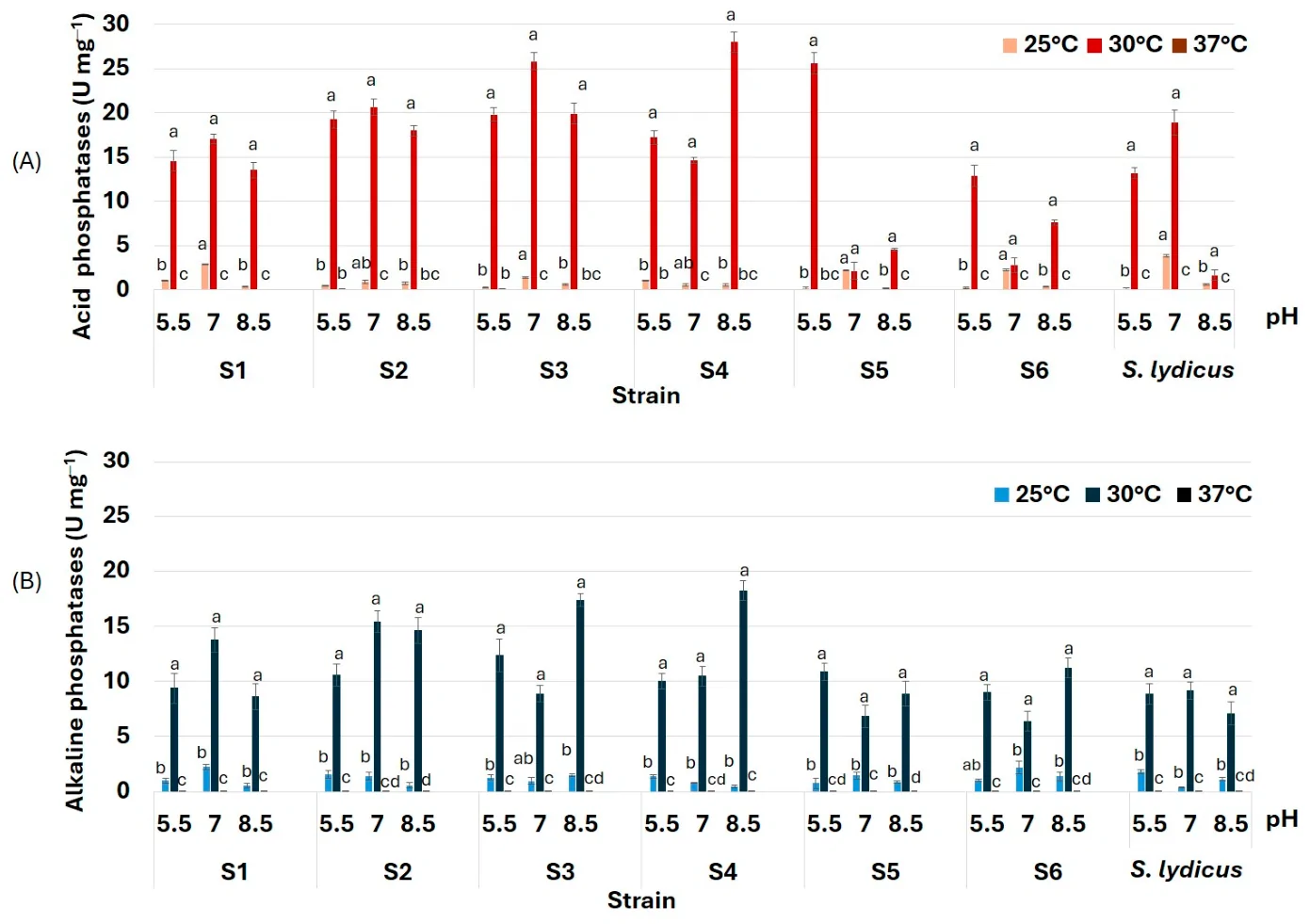
(**A**) Acid phosphatase activity; (**B**) Alkaline phosphatase activity, by strains S1–S6 in SP3 medium under different temperature and pH conditions. Bars labeled with different letters differ significantly (*p* < 0.05), as determined by pairwise comparisons of estimated marginal means using a generalized linear model with a Gamma distribution and log link; bars sharing at least one letter do not differ significantly.

Regarding pH, both enzyme activities increased significantly with pH, particularly when shifting from 5.5 (or 7.0, depending on the enzyme baseline) to 8.5 (alkaline phosphatases: Wald χ^2^ = 8.509, *p* = 0.014; acid phosphatases: Wald χ^2^ = 19.054, *p* < 0.001). With respect to strains, S2, S3, and S4 exhibited the highest production of both enzymes, with acid phosphatase activity approximately 23% and 29% higher than in the remaining strains. Significant differences among strains for both enzymatic activities are shown in Figure 3.

#### 3.4.4. Ammonium Production

Ammonia production was low in all strains, remaining below 0.025 U mg^−1^ protein. However, a generalized linear model (GLM) with a gamma distribution and log-link function revealed significant effects of temperature, pH, strain, and their two- and three-way interactions (*p* < 0.05). Dispersion diagnostics indicated adequate model fit (deviance/df = 0.335; Pearson χ^2^/df = 0.224).

**Figure 4:**
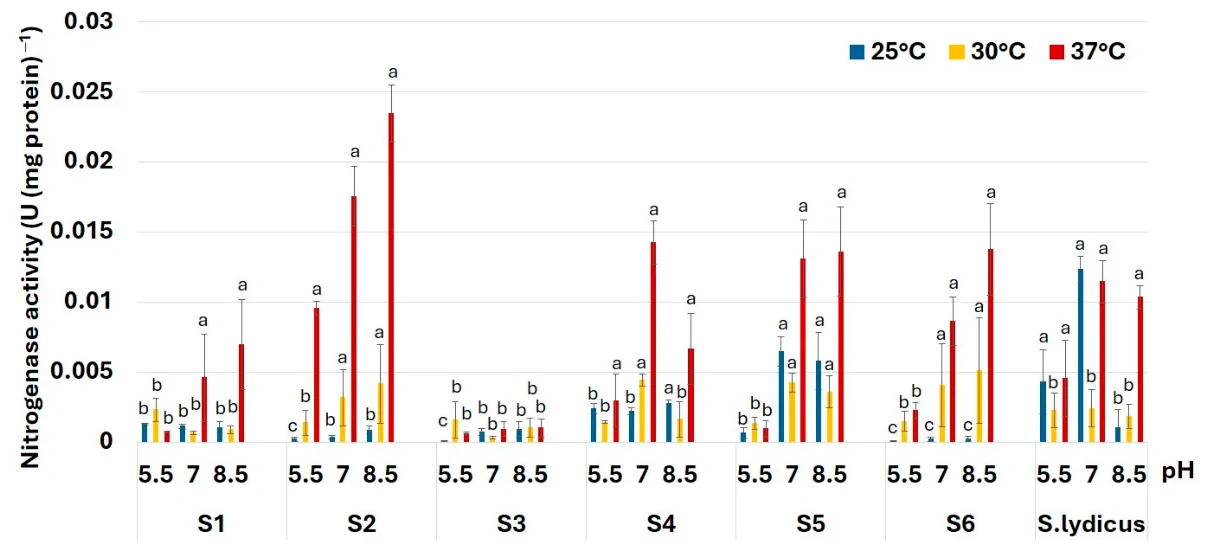
Nitrogenase activity in actinomycete strains grown under different temperature and pH conditions. Bars labeled with different letters differ significantly (*p* < 0.05), as determined by pairwise comparisons of estimated marginal means using a generalized linear model with a Gamma distribution and log link; bars sharing at least one letter do not differ significantly.

Temperature had the strongest effect on nitrogenase activity (Wald χ^2^ = 608.711, *p* < 0.001), followed by strain (χ^2^ = 494.195, *p* < 0.001) and the strain × temperature interaction (χ^2^ = 473.237, *p* < 0.001). Although pH was also significant (*p* < 0.001), its relative contribution was lower (χ^2^ = 190.472). These results indicate that temperature is the main determinant of nitrogenase activity, with strain-dependent responses (Figure 4). The highest nitrogenase activity was observed at 37 °C and pH 7.0–8.5. Regarding strain performance, nitrogenase activity followed the order: S2 > S5 ≈ *S. lydicus* > S4 ≈ S6 > S1 > S3.

#### 3.4.5. Salt Tolerance

A generalized linear model (GLM) with a gamma distribution and log-link function revealed significant effects of strain, salinity, and their interactions on microbial growth (*p* < 0.05). Dispersion diagnostics indicated deviance/df and Pearson χ^2^/df values of 0.050 and 0.049, respectively. Although both values were below 1, suggesting underdispersion, no evidence of lack of fit was detected.

**Figure 5:**
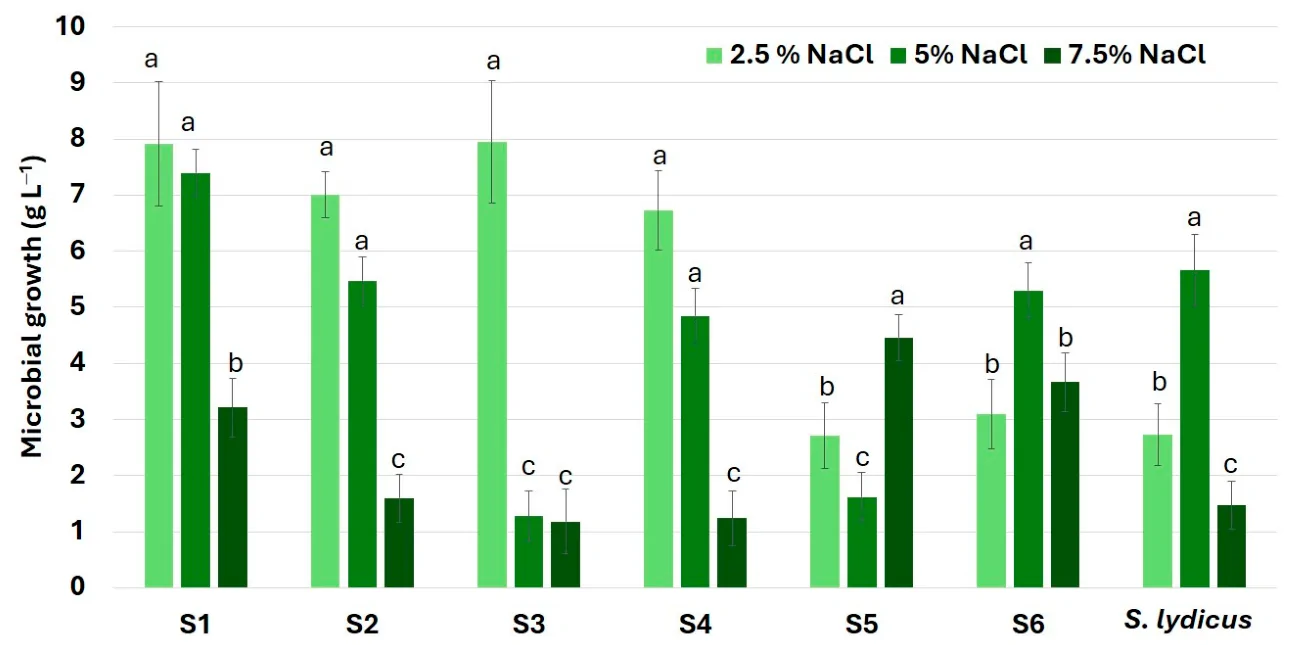
Salt tolerance of actinomycete strains. Bars labeled with different letters differ significantly (*p* < 0.05), as determined by pairwise comparisons of estimated marginal means using a generalized linear model with a Gamma distribution and log link; bars sharing at least one letter do not differ significantly.

The significant interaction effect indicates that the response of microbial growth to NaCl concentration was strain-dependent (Figure 5). Increasing the NaCl concentration from 2.5 to 7.5 g L^−1^ resulted in a reduction in growth for strains S1, S2, and S4. In contrast, strains S5 and S6 showed improved growth under saline conditions, indicating greater adaptation to salt stress. Despite the reduction observed in strain S1, its growth at the highest salinity level (7.5 g L^−1^) was comparable to that of strain S6, suggesting a substantial capacity to tolerate elevated salt concentrations. Overall, these results highlight marked differences among strains in their response to salinity and identify S1, S5, and S6 as the most promising candidates for growth under saline conditions.

### 3.5. Plant Growth-Promoting Functional Profile of the Strains

**Figure 6:**
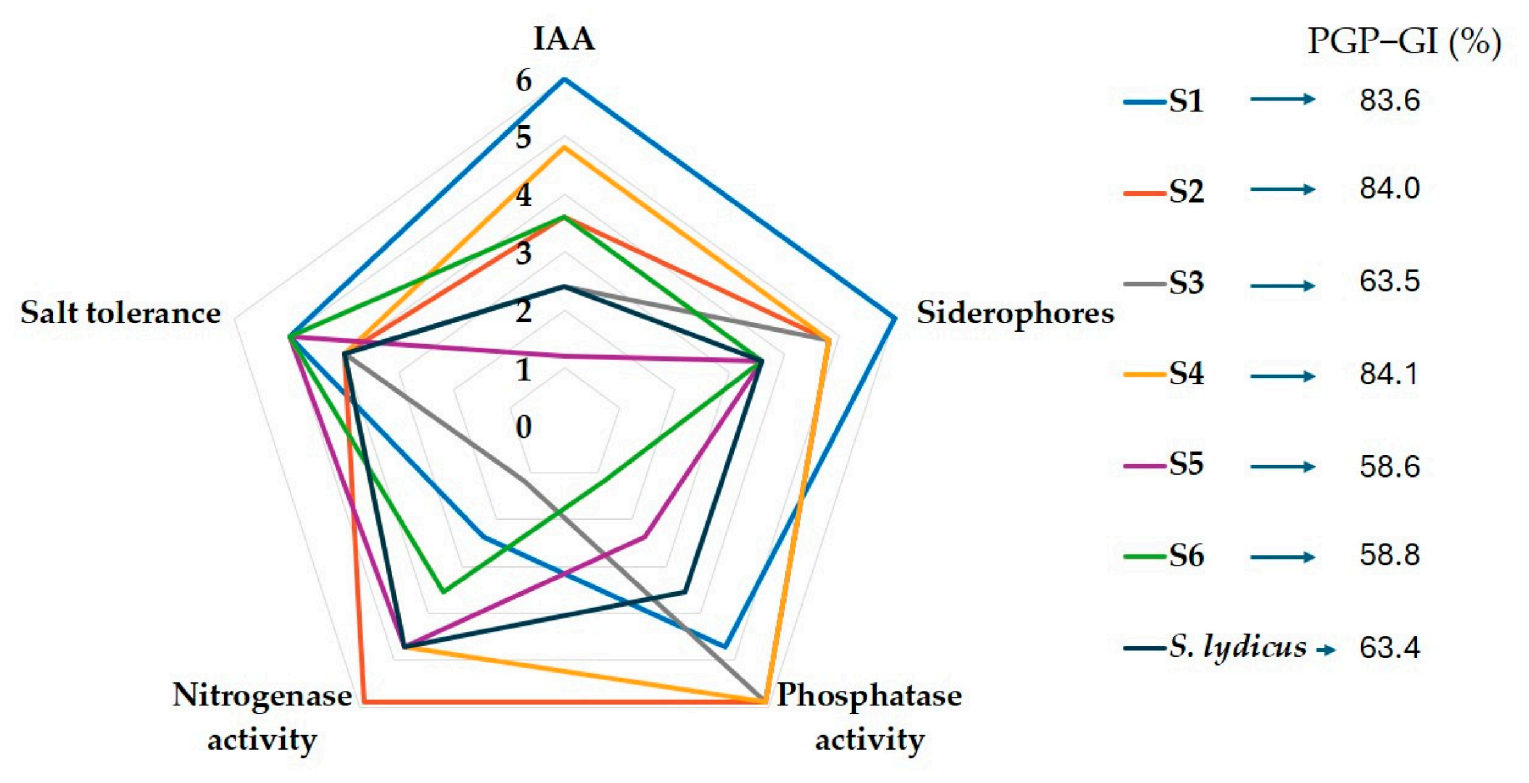
Radar plot comparison of the plant growth-promoting (PGP) functional profile of strains S1–S6, integrating indole-3-acetic acid (IAA) production, siderophore production, nitrogenase activity, phosphatase activity, and salt tolerance. The overall Plant Growth Promotion Global Index (PGP−GI) is indicated for each strain, with S4 (84.1%) and S1 (83.6%) showing the highest overall performance.

The Plant Growth Promotion Global Index (PGP-GI) values calculated for the evaluated strains are presented in Figure 6. Strain S4 exhibited the highest PGP-GI (84.1%), closely followed by strain S1 (83.6%), indicating superior overall performance compared with the remaining strains. For both strains, the production of PGP metabolites was generally favored at 30 °C and pH 7.0–8.5.

Radar plot analysis provided a comparative visualization of the physiological and biochemical profiles of the evaluated strains (Figure 6). Strain S1 was characterized by high indole-3-acetic acid (IAA) and siderophore production, although it exhibited comparatively low nitrogenase activity. Strains S2 and S4 showed high phosphatase and nitrogenase activities together with relatively high IAA and siderophore production. In contrast, strains S3, S5, and S6 displayed intermediate to low performance across most evaluated traits. Overall, all strains exhibited some degree of tolerance to saline conditions, although marked differences were observed in their responses to increasing NaCl concentrations.

### 3.6. Immobilization of Strains S1 and S4 in Dehydrated Alginate Matrices

The strains were propagated under the conditions identified as optimal for metabolite production. Cultivation was performed in ISP3 medium adjusted to the optimal pH for each strain (pH 7.0 for strain S1 and pH 8.5 for strain S4) and incubated at 30 °C. Cell viability was evaluated at each stage of the immobilization and dehydration process. Viability losses ranged between 2% and 12%, depending on the strain and processing step. The remaining viability at each stage, as well as the final viability after immobilization and dehydration, is summarized in Table 8. Statistical analysis using the Kruskal–Wallis test indicated no significant differences in viability loss between strains or processing stages (*p* > 0.05). Accordingly, the average overall viability loss across strains and process stages was 87 ± 3%.

**Figure 7:**
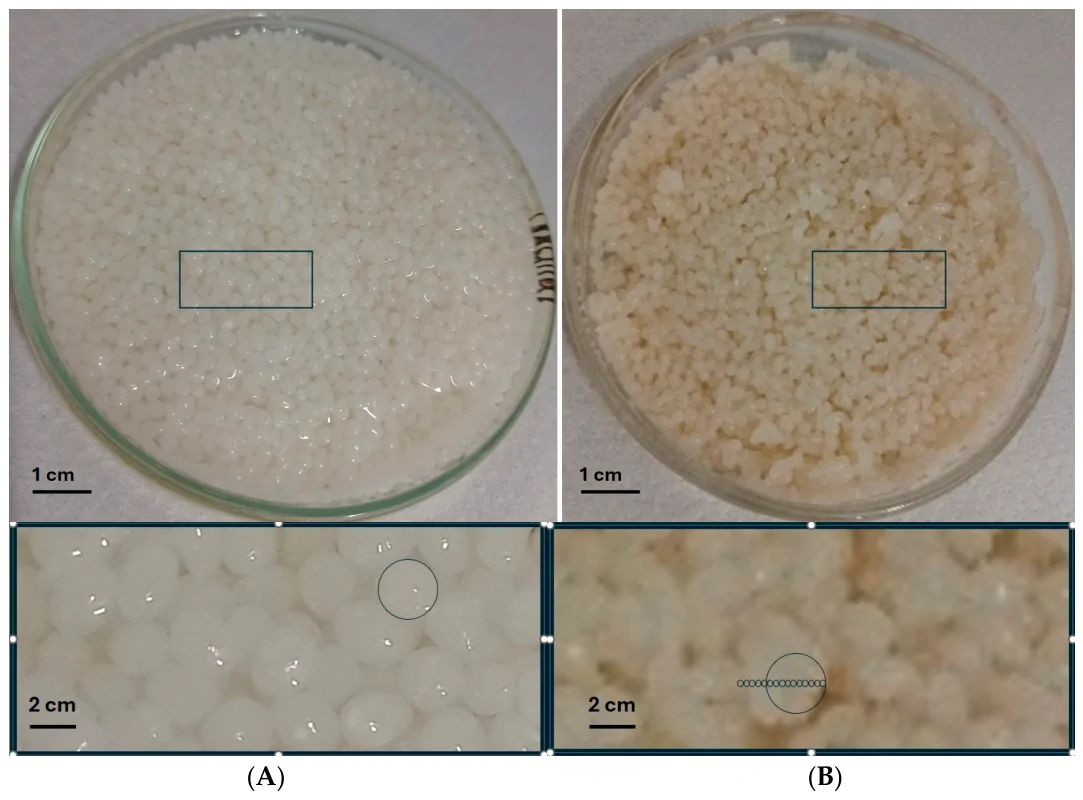
(**A**) S1 and (**B**) S4 strains immobilized in a calcium alginate matrix.

From an average mass of 33 ± 4.5 g of wet capsules, 1.8 ± 0.4 g of dehydrated capsules with an average diameter of 2.5 ± 0.2 mm were recovered after the dehydration process. The morphology of the calcium alginate capsules containing strains S1 and S4 is shown in Figure 7. Capsules containing strain S1 exhibited a white appearance, whereas capsules with strain S4 showed a yellowish coloration and an elastic, slightly adhesive texture. Differences in capsule appearance may be associated with the culture conditions used for each strain, particularly the pH of the growth medium (7.0 for S1 and 8.5 for S4). However, detailed structural characterization is required to confirm the potential effects of pH on matrix organization.

### 3.7. Tolerance of Strains S1 and S4 to 365 nm UV Irradiation

**Figure 8:**
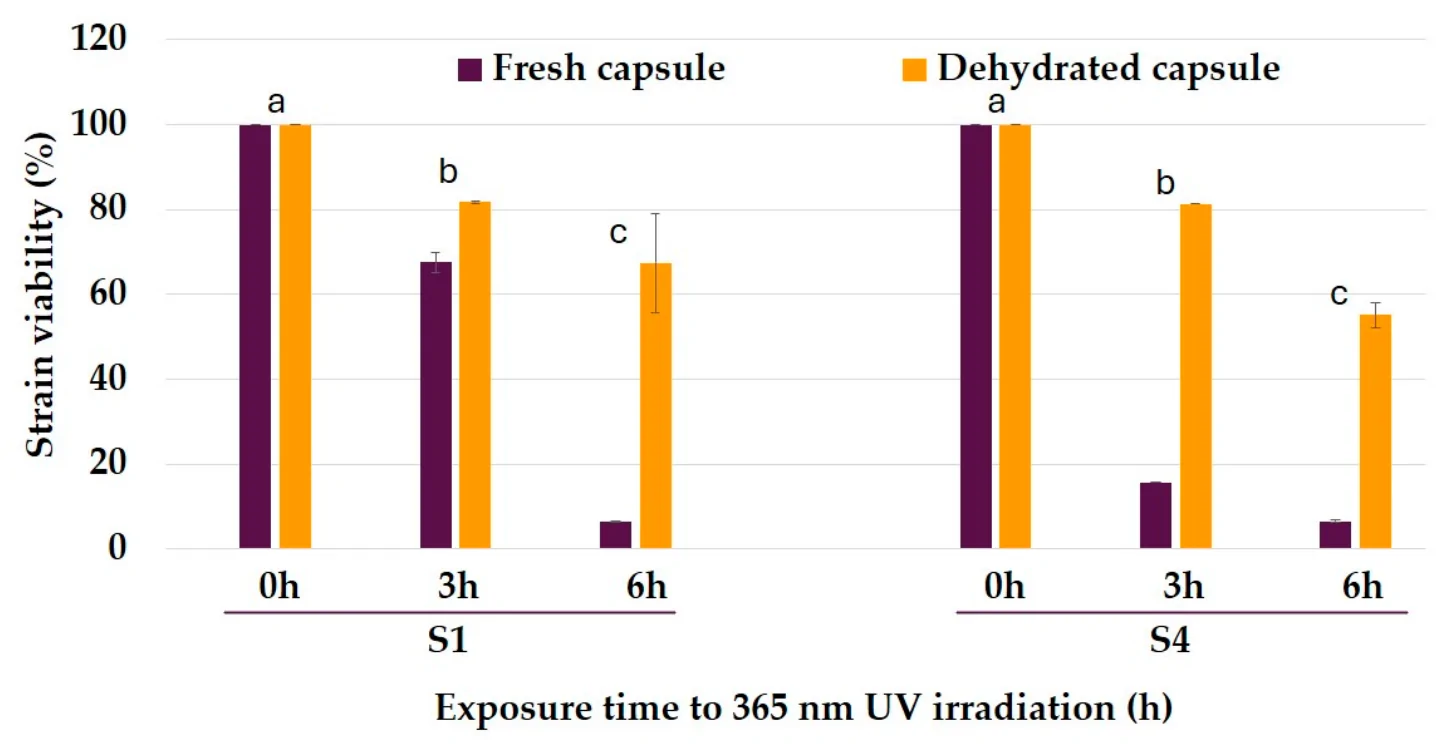
Remaining viability of strains S1 and S4 exposed to 365 nm UV irradiation in humid and dehydrated encapsulated formulations. Different letters denote significant differences according to the non-parametric Kruskal–Wallis test for a *p* < 0.05.

The viability of strains S1 and S4, encapsulated either as hydrated or dehydrated alginate capsules and exposed to 365 nm UV radiation for 3 and 6 h, is presented in Figure 8. Among the evaluated factors, only irradiation time had a significant effect on cell viability according to the non-parametric Kruskal–Wallis test (*p* < 0.05). Viability losses were substantially higher in hydrated capsules (approximately 93%) than in dehydrated capsules (33–45%). Overall, strain S1 exhibited greater resistance to UV irradiation than strain S4. This difference was particularly evident in the dehydrated formulations, in which the viability of strain S1 was approximately 12% higher after UV exposure.

### 3.8. Post-Encapsulation Metabolic Activity

Strains S1 and S4 were selected for encapsulation studies based on their contrasting physiological profiles in submerged culture. Strain S1 exhibited high production of indole-3-acetic acid (IAA; 25.15 ± 3.6 µg mL^−1^) and siderophores (67.50 ± 3.8 µg mL^−1^), whereas strain S4 was characterized by elevated enzymatic activities, including acid phosphatase (28.03 ± 1.2 U mg^−1^), alkaline phosphatase (18.23 ± 0.9 U mg^−1^), and nitrogenase (0.020 ± 0.004 U mg^−1^). These complementary traits justified their selection for evaluating the effects of encapsulation, dehydration, and UV exposure.

**Figure 9:**
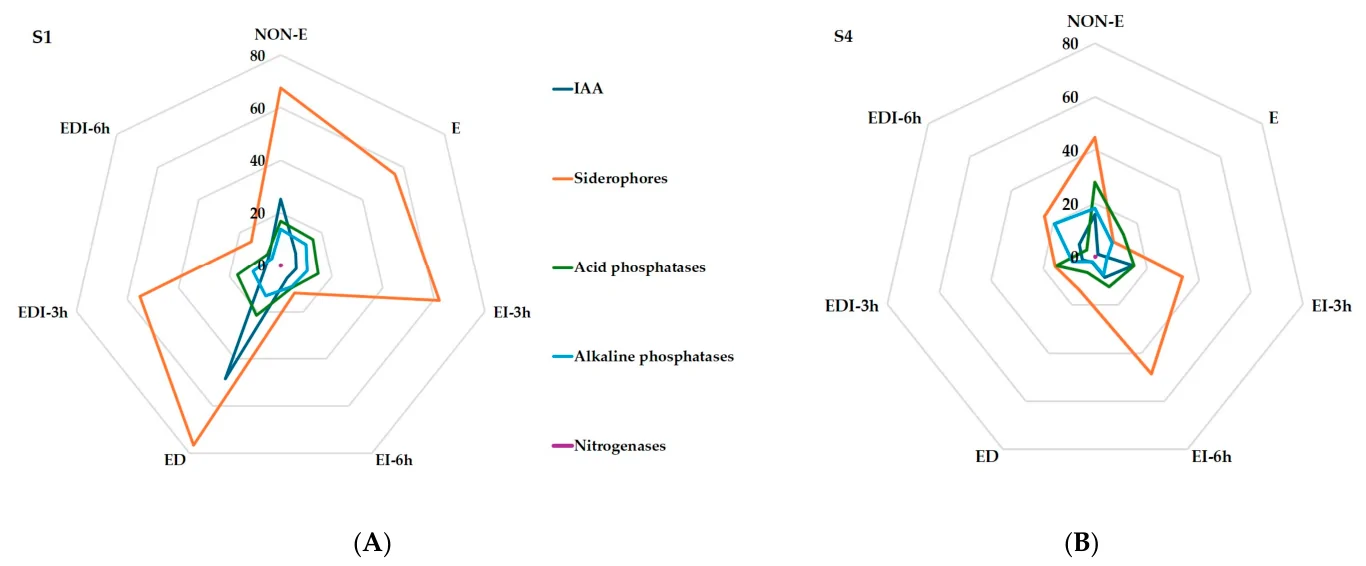
Metabolic and enzymatic activity of S1 (**A**) and S4 (**B**) strains after encapsulation, dehydration and irradiation treatments. NON-E: non-encapsulated (submerged culture); E: encapsulated; EI-3h: encapsulated and irradiated for 3 h; EI-6h: encapsulated and irradiated for 6 h; ED: encapsulated and dehydrated; EDI-3h: encapsulated, dehydrated and irradiated for 3 h; EDI-6h: encapsulated, dehydrated and irradiated for 6 h.

Encapsulation and dehydration affected the metabolic performance of the two strains differently (Figure 9). In strain S1, dehydration was associated with increased production of IAA (48.46 ± 3.8 µg mL^−1^) and siderophores (76.67 ± 3.8 µg mL^−1^) relative to the non-encapsulated control. However, prolonged UV exposure resulted in marked reductions in acid phosphatase activity (6.53 ± 0.6 U mg^−1^), alkaline phosphatase activity (4.19 ± 0.2 U mg^−1^), and released biomass (8.70 ± 0.3 g L^−1^). In contrast, strain S4 exhibited transient increases in siderophore production (48.71 ± 2.7 µg mL^−1^) and acid phosphatase activity (12.37 ± 0.6 U mg^−1^) when exposed to UV radiation in hydrated capsules. Nevertheless, dehydration substantially reduced its metabolic performance, particularly siderophore production (13.82 ± 0.4 µg mL^−1^) and acid phosphatase activity (6.53 ± 1.6 U mg^−1^). Although alkaline phosphatase activity remained detectable in dehydrated capsules exposed to UV radiation (19.66 ± 1.1 U mg^−1^), this response was not accompanied by similar preservation of the remaining functional traits.

Qualitative assays on solid media supported the trends observed in the quantitative analyses. Strain S1 maintained phosphate-solubilizing capacity after dehydration (PSI = 2.3–2.4) and showed partial recovery of cellulolytic activity under UV exposure (hydrolysis index ≈ 2.1). In contrast, strain S4 exhibited phosphate solubilization only under specific conditions, including UV-exposed hydrated capsules (PSI = 2.2 and 1.5), together with moderate cellulolytic activity (hydrolysis index = 1.33). Following dehydration, however, these activities were no longer detected. Overall, the results indicate greater tolerance of strain S1 to dehydration, whereas strain S4 showed a more variable physiological response to UV exposure. Comparison with the original submerged cultures further revealed that pre-encapsulation physiological performance did not necessarily predict post-encapsulation functionality, highlighting the importance of evaluating strain behavior under formulation and storage-related stresses.

## 4. Discussion

The propagation of six actinobacterial strains under different pH and temperature conditions, followed by encapsulation, dehydration, and UV exposure of selected strains, enabled assessment of the relationship between initial physiological performance and subsequent functionality. The results indicate that physiological plasticity constitutes an important component of biotechnological suitability, although high initial performance does not necessarily predict the ability to retain PGP functions after formulation.

Biomass production was strongly influenced by the interaction between temperature and pH, with maximum yields obtained at 37 °C (9.24 ± 1.06 g L^−1^) and pH 5.5 (8.91 ± 0.86 g L^−1^). Nevertheless, none of the isolates outperformed *Streptomyces lydicus*, used as the reference strain. Changes in pigmentation, pellet compaction, and differential release of hyphae and spores observed in both solid and liquid cultures suggest shifts in mycelial organization and metabolic resource allocation in response to environmental fluctuations, as previously described by Flärdh and Buttner (2009) [47].

The expression of PGP traits was highly dependent on environmental conditions. Production of indole-3-acetic acid (IAA), siderophores, phosphatases, growth in nitrogen-free medium, and hydrolytic activities were significantly affected by the interaction among strain, temperature, and pH, demonstrating pronounced functional plasticity, particularly for IAA and siderophore production (strain × pH × temperature interaction, *p* < 0.001). Strain S1 showed the highest levels of IAA production, reaching values 100–300% greater than those of the control strain under neutral and alkaline conditions at 30–37 °C. These values are comparable to those reported for rhizospheric actinomycetes, where IAA concentrations ranging from 11.40 ± 0.49 to 35.76 ± 2.92 μg mL^−1^ [48] and from 13.57 ± 0.32 to 23.61 ± 0.51 ppm [49] have been described. These observations suggest that certain strains possess a remarkable capacity to modulate phytohormone biosynthesis in response to environmental cues, a characteristic that may enhance their adaptability and performance in agricultural systems subjected to fluctuating soil conditions.

A similar trend was observed for siderophore production. Synthesis increased with increasing temperature and pH, reaching an average production of 38.6 μg mL^−1^ in strain S1 and maximum values of 67.50 ± 3.8 μg mL^−1^. The increase in siderophore production at alkaline pH is consistent with the reduced solubility and bioavailability of ferric iron under these conditions, a phenomenon widely recognized as stimulating iron-chelation mechanisms in microorganisms experiencing iron limitation [50,51]. These values are consistent with quantitative reports of hydroxamate-type siderophore accumulation using the same Atkin’s assay in other soil actinobacteria, such as *Nocardia mangyaensis* NH1 (87 ± 21 µM after 96 h [52]), confirming that the hydroxamate levels detected here fall within the range described for functionally active rhizospheric.

Phosphate solubilization and phosphatase production exhibited particularly interesting behavior. Strain S4 displayed the highest phosphate solubilization index among the evaluated isolates (PSI = 2.10), whereas alkaline and acid phosphatase activities increased approximately fifteen- and nine-fold, respectively, when the temperature increased from 25 to 30 °C. These findings suggest that moderate thermal conditions favor phosphorus mobilization mechanisms in these actinobacteria. Similar phosphate-solubilization indices have been reported in agricultural isolates exhibiting efficient phosphorus acquisition strategies [49]. Likewise, phosphatase activities recorded in strains S2, S3, and S4 were comparable to those reported for actinomycetes associated with mycorrhizae and agricultural rhizospheres [39].

Microbial growth in nitrogen-free medium and ammonium production followed patterns distinct from those observed for IAA and siderophores. Ammonium production remained below 0.025 U mg^−1^ protein in all strains, although significant effects of temperature, pH, and strain were detected, with maximum activity recorded at 37 °C and pH 7.0–8.5. The relatively low biological nitrogen fixation observed in this study is consistent with multitrait screening studies conducted on rhizospheric actinobacteria, in which nitrogenase activity and ammonium production were undetectable in all evaluated isolates [53].

Salt tolerance, particularly in strains S1, S5, and S6, allowed growth at NaCl concentrations as high as 7.5% (*w*/*v*), levels comparable to those reported for halotolerant PGP actinobacteria, including species of *Streptomyces* and *Kocuria* [44]. Although osmoprotective mechanisms were not investigated, this characteristic could confer adaptive advantages for application in saline agricultural soils.

Cellulolytic activity also depended strongly on previous physiological conditions. Maximum hydrolysis indices were observed at 30 °C, whereas activity was negligible at 25 °C. These findings are consistent with reports describing cellulolytic optima between 40 and 50 °C in different *Streptomyces* species [54,55], although active cellulase production under mesophilic conditions has also been documented in microorganisms from agricultural environments. In contrast, no degradation halos were observed in chitin-containing media despite evident microbial growth. These findings suggest that substrate utilization and extracellular chitinase production are independent physiological traits whose expression may require additional regulatory signals that are absent under the conditions evaluated.

Integration of all evaluated traits through the PGP-GI index summarized the functional complexity of the studied strains. Strains S4 (84.1%) and S1 (83.6%) exhibited the highest values and were therefore selected for formulation studies. However, their behavior after encapsulation, dehydration, and UV exposure demonstrated that the PGP-GI is not a direct predictor of technological resilience. Consequently, this index should be regarded as an indicator of physiological potential rather than a predictor of formulation robustness, highlighting the need to incorporate post-formulation functional stability into strain-selection schemes.

Encapsulation in calcium alginate followed by dehydration caused viability losses of only 10–14%, comparable to those reported for other dehydrated alginate-based microbial formulations [20]. Nevertheless, reductions in PGP traits suggest that encapsulation affected metabolite availability more strongly than cell survival. Although metabolite bioavailability was not directly evaluated, previous studies have shown that alginate matrices can modify mass transfer phenomena, extracellular metabolite diffusion, and physiological responses arising from microorganism–matrix interactions, including protection against UV radiation [21,56].

Following dehydration of encapsulated S1 cells, IAA and siderophore production increased to 48.46 ± 3.8 and 76.67 ± 3.8 μg mL^−1^, respectively, exceeding values obtained in submerged culture. Because physiological mechanisms associated with desiccation tolerance were not evaluated, these increases should be interpreted cautiously. Alternatively, they may reflect metabolite concentration effects, differential retention within the matrix, altered release patterns, or physiological responses induced by osmotic and desiccation stress. Similar phenomena have been reported previously, indicating that water loss can substantially modify microbial metabolism and extracellular metabolite recovery in a strain-dependent manner [13,21,57,58]. Alown et al. (2026) [13] also reported that environmental stress may alter resource allocation and secondary metabolite production in actinomycetes. In contrast, strain S4 retained only alkaline phosphatase activity after dehydration and UV exposure, suggesting that preservation of individual traits does not necessarily imply maintenance of the complete PGP profile.

Exposure of encapsulated strains to UV radiation provided an additional stress condition relevant to agricultural applications. Irradiation significantly decreased viability in fresh capsules, whereas dehydrated capsules retained 55–67% viability. In strain S1, prolonged exposure reduced IAA and siderophore production, whereas strain S4 exhibited transient increases in siderophore synthesis and selected enzymatic activities under specific exposure conditions. We propose, as a hypothesis for future investigation rather than an established mechanism, that UV-induced oxidative stress could promote Fe^2+^ oxidation to Fe^3+^, transiently increasing cellular iron demand and activating iron-dependent chelation pathways [23,59,60]. Testing this hypothesis would require molecular-level analyses—for example, transcriptional profiling of iron-homeostasis and oxidative-stress-response genes—that fall outside the scope of the present study, which was designed to characterize physiological and functional responses rather than their underlying regulatory mechanisms. Nevertheless, these findings demonstrate that responses to UV radiation depend on both the physiological state acquired during cultivation and the formulation condition.

One limitation of this study is that responses were evaluated only at physiological and functional levels, without molecular or transcriptomic analyses capable of identifying regulatory mechanisms associated with environmental preconditioning and formulation treatments. A further limitation is the use of n = 2 independent biological replicates per strain × pH × temperature combination; while sufficient to detect the large, consistent effects reported here, a larger number of replicates would increase statistical power to detect smaller interaction effects in future studies.

Finally, it remains necessary to determine whether the post-formulation functional resilience identified here translates into improved rhizosphere persistence and sustained agronomic benefits under cultivation conditions. Validation of this relationship would support the incorporation of functional resilience as an additional predictive criterion for the rational selection of actinobacteria intended for next-generation bioinoculant development.

## 5. Conclusions

This study demonstrates that pH and temperature are key modulators of the expression of plant growth-promoting (PGP) traits in native agricultural soil actinobacteria, with responses that are highly strain-specific and independent of biomass production. The significant strain × pH × temperature interaction observed for all quantitative PGP traits indicates that the functional potential of microbial candidates cannot be reliably assessed under a single cultivation condition. Furthermore, although strains S1 and S4 achieved similar PGP-GI values, they did so through distinct functional strategies, highlighting the importance of evaluating multiple mechanisms across environmental gradients to accurately identify robust microbial inoculants.

Encapsulation in calcium alginate effectively preserved cell viability, whereas the subsequent drying and UV exposure differentially affected metabolic functionality depending on the strain. The contrasting responses of S1 and S4 support the hypothesis that physiological plasticity under varying pH and temperature conditions is associated with the capacity to maintain PGP traits after formulation-related stress. More broadly, these results show that cell viability and functional performance are not equivalent outcomes of alginate encapsulation, underscoring the need to engineer and evaluate the polymeric matrix itself—not only the microbial strain—when designing formulations intended to retain plant growth-promoting activity after processing. Overall, these findings emphasize that integrating environmental preconditioning and post-processing functional stability into strain selection protocols may improve the development of more resilient and effective microbial bio-inputs for agricultural systems exposed to variable soil and climatic conditions.

## Figures and Tables

**Table 1:** Basal colonial morphology, radial growth, and phenotypic changes in actinomycetes cultured under different pH conditions (Czapek agar, 30 °C).

Strain	Colonial Morphology (pH 7.0)	Colonial Diameter (cm) *			Phenotypic Response to pH Change
		pH = 5.5	pH = 7.0	pH = 8.5	
S1	Circular, convex, powdery, white colony; reverse dark brown	0.6 ± 0.1	2.3 ± 0.1	0.6 ± 0.2	Synthesis and secretion of diffusible pigment into the medium induced at pH 8.5.
S2	Colony irregular, lobed, flat, beige color; brown reverse	1.2 ± 0.3	2.1 ± 0.4	1.2 ± 0.2	Focused chromogenic response with central dark pigmentation at pH 5.8.
S3	Circular colony, umbonate, dense in the center, beige in color; amber reverse	1.8 ± 0.3	2.4 ± 0.3	2.1 ± 0.2	Change in spore mass color at pH 8.5.
S4	Lobed, white colony with superficial spots; reverse amber with a brown margin.	1.0 ± 0.3	1.2 ± 0.3	1.0 ± 0.3	Highest diffusible pigment production at pH 7.0.
S5	Irregular, raised, beige to brown colony; reverse diffuse brown	0.5 ± 0.2	0.8 ± 0.2	0.9 ± 0.3	Radial growth increases linearly with pH
S6	Circular, lobed, convex, white colony; reverse light yellow	2.1 ± 0.3	2.5 ± 0.3	2.3 ± 0.3	Central dark pigmentation at pH 5.5

* Values represent the mean of two independent replicates ± standard deviation.

**Table 2:** Morphological characterization * of biomass of six actinobacterial strains cultivated in ISP3 medium under varying pH at 30 °C.

Strain	pH	Pellet Size	Density (Agglomeration)	Free Hyphae	Free Spores
S1	5.5	1	0	0	0
	7	1	0	0	1
	8.5	1	0	0	0
S2	5.5	1	0	0	2
	7	1	2	0	0
	8.5	2	2	0	0
S3	5.5	1	0	2	0
	7	1	0	3	0
	8.5	2	0	2	2
S4	5.5	3	0	0	2
	7	3	0	0	2
	8.5	3	0	2	2
S5	5.5	3	0	2	0
	7	0	0	0	3
	8.5	0	0	1	0
S6	5.5	2	0	2	2
	7	3	0	0	2
	8.5	1	0	2	0

* Note: Data were assigned based on direct microscopic observation using an ordinal scoring scale. Pellet size and density were classified as follows: 0 = absent; 1 = small/loose; 2 = moderate; 3 = large/compact. Release of hyphae and spores into the medium was scored as: 0 = absent; 1 = scarce, marginal presence in the field; 2 = moderate, covering up to 50% of the microscopic field; 3 = abundant, extensively occupying the field.

**Table 3:** Dry biomass production (g L^−1^) by actinomycete strains grown in ISP3 medium at different pH values and temperatures.

Temperature (°C)	pH	S1	S2	S3	S4	S5	S6
25	5.5	8.7 ± 1.2 ^bA^	9.3 ± 0.5 ^bA^	8.2 ± 1.3 ^bA^	7.8 ± 1.3 ^bA^	8.9 ± 1.4 ^bA^	8.8 ± 1.2 ^bA^
	7	7.9 ± 0.7 ^bAB^	7.6 ± 1.0 ^bAB^	8.3 ± 1.3 ^bAB^	7.2 ± 1.1 ^bAB^	7.9 ± 1.4 ^bAB^	8.8 ± 1.2 ^bAB^
	8.5	8.6 ± 0.6 ^bB^	6.9 ± 0.4 ^bB^	8.5 ± 1.7 ^bB^	7.2 ± 0.5 ^bB^	7.3 ± 1.1 ^bB^	6.7 ± 1.5 ^bB^
30	5.5	9.4 ± 0.8 ^bA^	8.9 ± 0.6 ^bA^	9.0 ± 0.3 ^bA^	9.4 ± 0.4 ^bA^	7.9 ± 1.0 ^bA^	9.0 ± 0.5 ^bA^
	7	8.6 ± 1.0 ^bAB^	7.2 ± 1.2 ^bAB^	8.7 ± 1.1 ^bAB^	8.5 ± 1.5 ^bAB^	9.0 ± 1.0 ^bAB^	8.5 ± 0.9 ^bAB^
	8.5	7.9 ± 1.2 ^bB^	8.3 ± 1.4 ^bB^	7.8 ± 1.3 ^bB^	8.6 ± 1.6 ^bB^	8.6 ± 1.3 ^bB^	9.0 ± 1.2 ^bB^
37	5.5	9.1 ± 0.8 ^aA^	9.2 ± 0.5 ^aA^	9.7 ± 1.0 ^aA^	8.6 ± 1.5 ^aA^	8.8 ± 0.8 ^aA^	9.7 ± 0.8 ^aA^
	7	10.1 ± 1.0 ^aAB^	9.2 ± 1.6 ^aAB^	8.9 ± 0.4 ^aAB^	9.4 ± 1.4 ^aAB^	9.6 ± 0.8 ^aAB^	11.5 ± 1.1 ^aAB^
	8.5	9.0 ± 0.6 ^aB^	9.1 ± 0.9 ^aB^	8.1 ± 0.6 ^aB^	8.5 ± 1.4 ^aB^	8.9 ± 1.3 ^aB^	9.0 ± 1.6 ^aB^

Note: Three-way ANOVA. Different letters indicate significant differences according to Tukey’s test (*p* < 0.05). Lowercase letters denote differences among temperature levels, whereas uppercase letters denote differences among pH levels.

**Table 4:** Ordinal scores of microbial growth * of the strains in nitrogen-free medium.

Strain	pH	25 °C	30 °C	37 °C
S1 ^bc^	5.5	2	2	1
	7	2	2	0
	8.5	2	2	1
S2 ^ab^	5.5	2	2	2
	7	2	3	3
	8.5	2	2	2
S3 ^ab^	5.5	2	2	2
	7	3	3	1
	8.5	2	2	1
S4 ^a^	5.5	3	3	2
	7	3	2	2
	8.5	3	2	2
S5 ^c^	5.5	1	1	0
	7	1	2	0
	8.5	0	2	0
S6 ^bc^	5.5	2	2	1
	7	2	2	0
	8.5	2	2	1
*S. lydicus* ^ab^	5.5	1	3	2
	7	2	3	2
	8.5	2	0	2

* Microbial growth was scored ordinally via direct microscopic observation: 0 = none; 1 = low (0 < colonies ≤ 5 mm); 2 = medium (5 mm ≤colonies ≤ 10 mm); 3 = abundant (colonies ≥ 10 mm). Different letters indicate significant differences according to the Mann–Whitney test and Bonferroni correction to control for errors due to multiple comparisons (α = 0.005).

**Table 5:** Phosphate solubilization index * (PSI) of actinomycete strains on Pikovskaya medium following inoculum production under different pH and temperature conditions in ISP3 medium.

Strain	pH	25 °C	30 °C	37 °C
S1 ^cd^	5.5	1.14	0	≤1
	7.0	1.14	0	≤1
	8.5	1.14	0	≤1
S2 ^d^	5.5	≤1	≤1	0
	7.0	≤1	≤1	0
	8.5	≤1	≤1	0
S3 ^c^	5.5	1.14	1.3	≤1
	7.0	1.14	1.3	0
	8.5	1.14	1.1	0
S4 ^b^	5.5	2.00	1.4	1.2
	7.0	2.10	1.8	1.2
	8.5	1.60	1.5	1.2
S5 ^e^	5.5	0	0	0
	7.0	0	0	≤1
	8.5	0	≤1	0
S6 ^d^	5.5	1.14	≤1	0
	7.0	≤1	≤1	0
	8.5	1.14	≤1	0
*S. lydicus* ^a^	5.5	2.20	1	1
	7.0	2.60	1	1
	8.5	2.00	1	1

* Phosphate solubilization index (PSI): PSI ≤ 1.0, no activity; 1.5 ≤ PSI < 2.0, low activity; 2.0 ≤ PSI < 3.0, moderate activity; 3.0 ≤ PSI, high activity; PSI = 0, no microbial growth. Different letters indicate statistically significant differences among estimated marginal means (*p* < 0.05).

**Table 6:** Cellulolytic hydrolysis index * of strains grown on carboxymethylcellulose-containing solid medium.

Strain	pH	25 °C	30 °C	37 °C
S1	5.5	1	>2	>2
	7.0	1	>2	>2
	8.5	1	>2	>2
S2	5.5	1 > IH ≤ 2	>2	1 > IH ≤ 2
	7.0	1 > IH ≤ 2	>2	1 > IH ≤ 2
	8.5	1 > IH ≤ 2	>2	1 > IH ≤ 2
S3	5.5	1	>2	>2
	7.0	1	>2	>2
	8.5	1	>2	>2
S4	5.5	1	>2	>2
	7.0	1	>2	1
	8.5	1	>2	>2
S5	5.5	1	1 > IH ≤ 2	1 > IH ≤ 2
	7.0	1	1 > IH ≤ 2	1 > IH ≤2
	8.5	1	1 > IH ≤ 2	1 > IH ≤ 2
S6	5.5	1	>2	>2
	7.0	1	>2	1
	8.5	1	>2	>2
*S. lydicus*	5.5	1	1 > IH ≤ 2	1 > IH ≤ 2
	7.0	1	1 > IH ≤ 2	1 > IH ≤ 2
	8.5	1	1 > IH ≤ 2	1 > IH ≤ 2

* Hydrolysis index (HI) in medium with CMC. HI = 1, no activity; 1 < HI < 2, moderate enzymatic activity; HI ≥ 2, good enzymatic activity.

**Table 7:** Growth of actinomycetes on colloidal chitin (1%)-supplemented agar at 25, 30, and 37 °C.

Strain	pH	25 °C	30 °C	37 °C
S1 ^bc^	5.5	1	1.1	Ncg *
	7.0	0.8	1.3	ncg
	8.5	1	1.3	ncg
S2 ^a^	5.5	1.1	1.5	1
	7.0	1.3	1.6	1.5
	8.5	1.1	1.5	0.8
S3 ^ab^	5.5	1.1	1.2	1.3
	7.0	1	1.2	1.2
	8.5	1.2	2	0.7
S4 ^ab^	5.5	ncg	1.2	1
	7.0	ncg	1.3	1.2
	8.5	ncg	1.3	1.4
S5 ^c^	5.5	ncg	ncg	ncg
	7.0	1	ncg	ncg
	8.5	ncg	0.6	ncg
S6 ^bc^	5.5	1.1	1.5	ncg
	7.0	0.8	1.3	ncg
	8.5	1	0.8	ncg
*S. lydicus* ^bc^	5.5	ncg	0.5	ncg
	7	ncg	1.5	1.3
	8.5	ncg	0.6	1.3

* Note: ncg = No colony growth. Different letters indicate statistically significant differences among estimated marginal means (*p* < 0.05).

**Table 8:** Viability (%) of strains S1 and S4 after each processing stage (dispersion, immobilization, and dehydration) and remaining viability *.

Strain	Dispersion in Sodium Alginate	Immobilization in Calcium Alginate	Dehydration of Immobilized Strains	Remaining Viability
S1	97.8 ± 3.5	97.4 ± 0.6	93.2 ± 0.7	88.8 ± 3.3
S4	93.8 ± 1.9	92.6 ± 6.4	98.5 ± 1.6	85.4 ± 2.9

* The reported values are the average of two replicates. The non-parametric Kruskal–Wallis test showed no significant differences (*p* < 0.05), either by strain type or by process stage.

## Data Availability

The original contributions presented in the study are included in the article; further inquiries can be directed to the corresponding author/s.

## Supplementary Materials

The following supporting information can be downloaded at: https://www.mdpi.com/article/10.3390/polym18172041/s1, Table S1. Colony morphology and molecular identification of the six native actinobacterial strains characterized in this study.

## Abbreviations

The following abbreviations are used in this manuscript:

CMC	Carboxymethylcellulose
CFU	Colony-forming units
GLM	Generalized linear model
HI	Hydrolysis index
IAA	indole-3-acetic acid
NFB	Nitrogen-free bromothymol blue
PGP	Plant growth-promoting
PGP−GI	Plant Growth-Promoting Global Index
PGP−Ms	Plant growth-promoting microorganisms
PSI	Phosphate solubilization index
S1, S2, S3, S4, S5, S6	Native strains of an agricultural soil
Si	Normalized score corresponding to each trait
UV	Ultraviolet
w_i_	Weight assigned to each trait
