# Supplementary material for: Environmental Preconditioning Shapes the Expression and Post-Formulation Stability of Plant Growth-Promoting Traits in Native Actinobacteria
Source: Polymers (Basel). 2026 Aug 22;18(17):2041. doi: 10.3390/polym18172041 (PMC13568018; doi:10.3390/polym18172041)
Supplement: Supplementary file 1 [file polymers-18-02041-s001.zip › polymers-4447752-supplementary.pdf]

Supplementary Table S1. Colony morphology and molecular identification of the six native actinobacterial strains characterized in this study. Colonies were grown on ISP2 agar for 7 days at 30 °C prior to photography. Molecular identification was performed by 16S rRNA gene sequencing and confirmed the genus-level identity of strains S2–S6 as *Streptomyces* sp.; GenBank accession numbers are indicated for each strain. Identification of strain S1 is in progress following a contamination event that required re-isolation prior to DNA extraction.

Strain	Colony morphology (7 d, ISP2 agar)	Molecular identification (16S rRNA)
**S1**	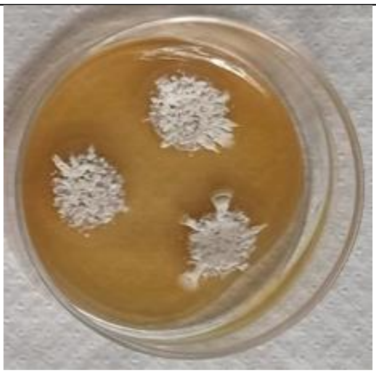	Identification in progress<br><br>(re-isolation following contamination)
**S2**	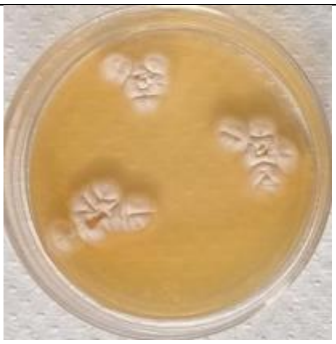	ID: *Streptomyces* sp.<br><br>GenBank accession: PZ784602
**S3**	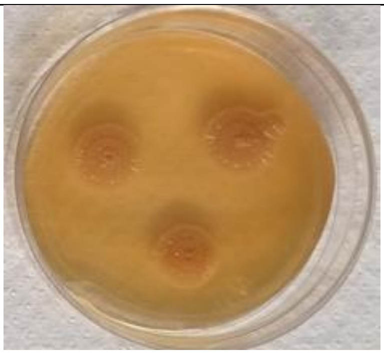	ID: *Streptomyces* sp.<br><br>GenBank accession: PZ784603

**S4**	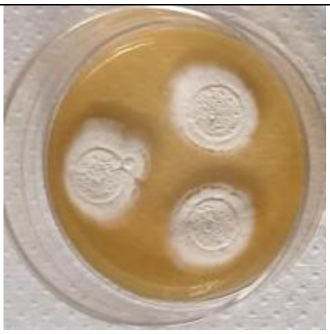	ID: *Streptomyces* sp.<br><br>GenBank accession: PZ784604
**S5**	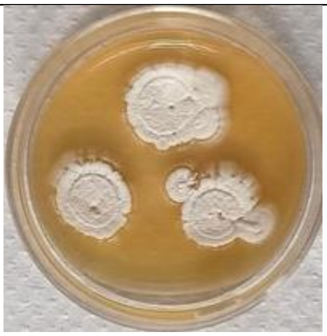	ID: *Streptomyces* sp.<br><br>GenBank accession: PZ784605
**S6**	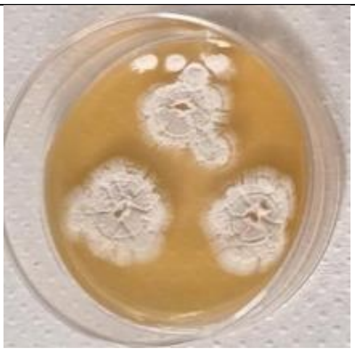	ID: *Streptomyces* sp.<br><br>GenBank accession: PZ784606
